# Theory of optical tweezing of dielectric microspheres in chiral host media and its applications

**DOI:** 10.1038/s41598-020-73530-1

**Published:** 2020-10-05

**Authors:** Rfaqat Ali, Rafael S. Dutra, Felipe A. Pinheiro, Felipe S. S. Rosa, Paulo A. Maia Neto

**Affiliations:** 1 Instituto de Física, Universidade Federal do Rio de Janeiro, Caixa Postal 68528, Rio de Janeiro, RJ 21941-972 Brasil; 2 LISComp-IFRJ, Instituto Federal de Educação, Ciência e Tecnologia, Rua Sebastião de Lacerda, Paracambi, RJ 26600-000 Brasil; 3 Applied Physics Department, Photonics Research Center, Gleb Wataghin Physics Institute, University of Campinas - UNICAMP, P.O. Box 6165, Campinas, SP 13083-970 Brazil

**Keywords:** Optical manipulation and tweezers, Nanophotonics and plasmonics

## Abstract

We report for the first time the theory of optical tweezers of spherical dielectric particles embedded in a chiral medium. We develop a partial-wave (Mie) expansion to calculate the optical force acting on a dielectric microsphere illuminated by a circularly-polarized, highly focused laser beam. When choosing a polarization with the same handedness of the medium, the axial trap stability is improved, thus allowing for tweezing of high-refractive-index particles. When the particle is displaced off-axis by an external force, its equilibrium position is rotated around the optical axis by the mechanical effect of an optical torque. Both the optical torque and the angle of rotation are greatly enhanced in the presence of a chiral host medium when considering radii a few times larger than the wavelength. In this range, the angle of rotation depends strongly on the microsphere radius and the chirality parameter of the host medium, opening the way for a quantitative characterization of both parameters. Measurable angles are predicted even in the case of naturally occurring chiral solutes, allowing for a novel all-optical method to locally probe the chiral response at the nanoscale.

One of the landmarks in the field of optomechanics was the advent of optical tweezers^1^, that allows for manipulation of microsized dielectric spheres and nanostructures trapped by a single tightly focused laser beam. Several applications in cell^2–4^ and molecular biology^5,6^, chemistry^7^, nanotechnology^8^ and physics^9–12^ have been developed (for reviews see^13–16^). The physical phenomena behind the operation of optical tweezers rely on momentum conservation as the incident trapping beam interacts with the microsphere. On one hand, within the geometrical optics approximation, the refracted light rays provide the key contribution to the optical force, which points towards the focal point, whereas reflected rays provide a detrimental radiation pressure contribution^17^. On the other hand, the Mie-Debye theory accounts for the exact wave-optical redistribution^18–20^ of linear momentum engendered by Mie scattering at the trapped microsphere. Playing with directional scattering in a metamaterial platform allows for trapping of high-index microspheres^21^.

In addition to linear momentum, a circularly-polarised (CP) light beam also carries spin angular momentum (SAM) that can be transferred to trapped particles^22–25^. The resulting optical torque leads to a rotation of the equilibrium position when a Stokes drag force is simultaneously applied^26^. The optical force on chiral particles has been employed for chirality sorting and recognition in optical traps^27–30^. Enantioselective optical manipulation schemes have been proposed^31–34^, including the possibility of employing optical torques^35,36^. The transfer of optical angular momentum dramatically changes when trapping particles in a chiral host medium^37^.

In this paper, we develop the theory of optical tweezers in a chiral medium. Our approach is based on the full Mie scattering solution for spherical particles embedded in a chiral medium^38^, combined with a Debye-type non-paraxial vector model for the trapping beam^39^. We consider a CP trapping beam and calculate the optical torque resulting from the transfer of SAM to the trapped particle. When a lateral Stokes drag force is simultaneously applied^26,36^, the equilibrium position is displaced sideways and rotates around the optical axis as a consequence of the optical torque. The angle of rotation is strongly chiral-dependent and very large when considering radii a few times larger than the wavelength.

Chirality is a geometrical property of structures including biological molecules^40^, random arrangement of plasmonic nanostructures^41,42^ and nanocrystals^43^ that are not superposable with their mirror objects^44,45^. Chirality plays an essential role in several biological, chemical and nanotechnological applications^46^. Our results for the particle rotation open the way for the characterization of the local chiral response at the nanoscale, that nicely interconnects with already existing local probing techniques to determine viscoelastic properties^47,48^ and micro-rheological properties of small particles^49–52^.

The optical force and torque are strongly dependent on the handedness of the CP trapping laser beam, as expected since the two different helicities propagate according to different refraction indexes in the chiral host medium. Our results indicate that a chiral host medium allows for trapping of high-index particles, and more generally improves the trap axial stability, provided that the CP laser beam and the host medium have the same handedness.

## Results

### Electromagnetic fields in chiral medium

The constitutive relations for a chiral medium contain a direct coupling between the electric field ${\mathbf{E}}$ and the auxiliary field ${\mathbf{H}}$ proportional to the chirality parameter $\kappa .$ They connect the complex displacement field ${\mathbf{D}}$ and magnetic field ${\mathbf{B}}$ to ${\mathbf{E}}$ and ${\mathbf{H}}$ as follows^53–56^:

1 $$\begin{aligned} \left[ {\begin{array}{*{20}l} {\mathbf{D}} \\ {\mathbf{B}} \\ \end{array} } \right] = \left[ {\begin{array}{*{20}l} {\varepsilon _0 \varepsilon } &{} {\quad i \sqrt{\varepsilon _0 \mu _0} \, \kappa } \\ {-i\sqrt{\varepsilon _0 \mu _0} \,\kappa } &{} {\quad \mu _0 \mu } \\ \end{array} } \right] \left[ {\begin{array}{*{20}l} {\mathbf{E}} \\ {\mathbf{H}} \\ \end{array} } \right] \end{aligned}$$

where $\varepsilon$ and $\mu$ are the relative permittivity and relative permeability of the medium, respectively. The constant $\kappa$ is the chirality parameter that characterizes the strength of chirality and usually satisfies the condition $\kappa \ll \sqrt{\varepsilon \mu }$. By using the aforementioned constitutive relations the Maxwell’s equations for a chiral medium in the frequency domain (frequency $\omega$) can be written in matrix form as

2 $$\begin{aligned}&\varvec{\nabla } \cdot \left[ {\begin{array}{*{20}l} {\mathbf{E}}\\ {\mathbf{H}} \end{array} } \right] = 0 \end{aligned}$$

3 $$\begin{aligned}&\varvec{\nabla } \times \left[ {\begin{array}{*{20}l} {\mathbf{E}}\\ {\mathbf{H}} \end{array} } \right] - K \left[ {\begin{array}{*{20}l} {\mathbf{E}} \\ {\mathbf{H}} \end{array} } \right] =0 \end{aligned}$$

where

4 $$\begin{aligned} K= \left[ {\begin{array}{ll} k_0 \kappa &{} i k_0 \mu \sqrt{\frac{\mu _0}{\varepsilon _0}} \\ -ik_0 \varepsilon \sqrt{\frac{\varepsilon _0}{\mu _0}} &{}\quad k_0 \kappa \end{array} } \right] \end{aligned}$$

and $k_0=\omega /c.$ The propagation modes are obtained by diagonalizing the matrix *K* through a linear transformation^53,57^

5 $$\begin{aligned} \left[ {\begin{array}{*{20}l} {\mathbf{E}}\\ {\mathbf{H}} \end{array} } \right] = \left[ {\begin{array}{ll} 1&{}\quad {-i \mu }/{\varepsilon }\\ {-i \varepsilon }/{\mu }&{}\quad 1 \end{array} } \right] \left[ {\begin{array}{*{20}l} {\mathbf{Q}}_+ \\ {\mathbf{Q}}_- \end{array} } \right] . \end{aligned}$$

${\mathbf{Q}}_+$ and ${\mathbf{Q}}_-$ independently satisfy the Helmholtz equation

6 $$\begin{aligned} \nabla ^{2}{} {\mathbf{Q}}_{\sigma }+k_{\sigma } ^{2}{} {\mathbf{Q}}_{\sigma }=0 \end{aligned}$$

and the subsidiary equations

7 $$\begin{aligned} \nabla \times {\mathbf{Q}}_{\sigma } + k {\mathbf{Q}}_{\sigma }=0 \quad , \quad \nabla \cdot {\mathbf{Q}}_{\sigma }=0 , \end{aligned}$$

with $\sigma =\pm 1$ representing helicity. The corresponding wavenumbers are given by

8 $$\begin{aligned} k_{\sigma } = k_0 (\sqrt{\varepsilon \mu }+\sigma \kappa ) . \end{aligned}$$

Thus, the propagation of a mode of helicity $\sigma$ is governed by the refractive index $n_{\sigma }= \sqrt{\varepsilon \mu }+\sigma \kappa .$

Finally, for future reference, we define the Debye potentials $\Pi ^{\mathrm{E}}$ and $\Pi ^{\mathrm{M}}$ for electric (E) and magnetic (M) multipoles, respectively^58,59^:

9 $$\begin{aligned} \begin{aligned} {\mathbf{E}}= \nabla \times \nabla \times \left( {\mathbf{r}} \Pi ^{\mathrm{E}}\right) +i\omega \mu \mu _{0} \nabla \times \left( {\mathbf{r}} \Pi ^{\mathrm{M}}\right) ,\\ {\mathbf{H}}= \nabla \times \nabla \times \left( {\mathbf{r}}\Pi ^{\mathrm{M}} \right) -i\omega \varepsilon \varepsilon _0 \nabla \times \left( {\mathbf{r}}\Pi ^{\mathrm{E}}\right) . \end{aligned} \end{aligned}$$

### The electromagnetic stress tensor in a chiral medium

When a light beam is scattered off a particle, it imparts an optical force on it due to momentum conservation. Such force may be calculated by integrating the Maxwell stress tensor $\overset{\leftrightarrow }{\mathbf{T}}$ over a closed Gaussian surface *S* that wraps around the particle:

10 $$\begin{aligned} {\mathbf{F}}= \oint _S \langle \overset{\leftrightarrow }{\mathbf{T}} \rangle \cdot {\mathbf {dA}}. \end{aligned}$$

As the stress tensor is quadratic in the electromagnetic fields, the following identity^60^ is useful when evaluating the time average $\langle ... \rangle$ in (10):

11 $$\begin{aligned} \langle \pmb {\mathscr {V}}_1({\mathbf{r}},t) \, \pmb {\mathscr {V}}_2({\mathbf{r}},t) \rangle = \frac{1}{2} \,{\mathrm{Re}} \, \left[ {\mathbf{V}}_1 ({\mathbf{r}}) \, {\mathbf{V}}_2^*({\mathbf{r}}) \right] \end{aligned}$$

where $$\pmb {\mathscr {V}}_j= {\mathrm{Re}}({\mathbf{V}}_j\,e^{-i\omega t})$$$$j=1,2$$ are general monocromatic fields.

We evaluate the surface integral in (10) for a Gaussian spherical surface *S*(*R*) of radius *R* centered at the origin. Using (11) and taking the standard explicit expression for the stress tensor $\overset{\leftrightarrow }{\mathbf{T}}$ in a non-viscous, incompressible liquid at rest^61,62^, we find

12 $$\begin{aligned} {\mathbf{F}}= {} \frac{R^2}{4}\oint _{S(R)}d\Omega \; {\mathrm{Re}} \Bigl [{\mathbf{E}}\left( {\mathbf{D}}^{*}\!\! \cdot \hat{\mathbf{r}}\right) + {\mathbf{D}}\left( {\mathbf{E}}^{*}\!\! \cdot \hat{\mathbf{r}}\right) +\, {\mathbf{H}}\left( {\mathbf{B}}^{*}\!\! \cdot \hat{\mathbf{r}}\right) + {\mathbf{B}}\left( {\mathbf{H}}^{*}\!\! \cdot \hat{\mathbf{r}}\right) - ({\mathbf{E}} \cdot {\mathbf{D}}^{*}+{\mathbf{H}} \cdot {\mathbf{B}}^{*}) \hat{\mathbf{r}} \Bigr ]. \end{aligned}$$

Note that there is a disagreement between the Minkowski and Abraham prescriptions, but it manifests itself in the momentum density, not in the momentum flux, so the stress tensor is the same. Finally, replacing the constitutive relations (1) for a non-magnetic chiral medium into (12) and taking $R \rightarrow \infty$, we arrive at

13 $$\begin{aligned} {\mathbf{F}} = -\lim _{R\rightarrow \infty }\frac{R^2}{2} \int _{S(R)} \!\!\! d\Omega \, \hat{\mathbf{r}} \biggl [\frac{\varepsilon _0\varepsilon \mathbf{E\cdot E^{*}} + \mu _0 {\mathbf{H\cdot H^{*}}}}{2} - \sqrt{\varepsilon _{0}\mu _{0}}\, \kappa \, \mathrm{Im}({\mathbf {E}}^{*}\cdot {\mathbf {H}}) \biggr ] , \end{aligned}$$

where we have used that the radial field components decay as $1/r^2$ and hence do not contribute to the flux.

### Theory of optical tweezers of a dielectric sphere embedded in a chiral medium

Here we combine the previous results in order to develop a generalization of the Mie-Debye theory to the case of a chiral host medium. We consider a CP Gaussian laser beam of helicity $\sigma =\pm 1$ at the entrance port of a high-numerical aperture (NA) objective. The resulting non-paraxial focused beam propagates in the lossless non-magnetic chiral medium of refractive index $n_{\mathrm{m}}(\sigma )=\sqrt{\varepsilon _{{\mathrm{m}}}}+\sigma \kappa$, where $\varepsilon _{{\mathrm{m}}}$ is the relative permittivity and $\kappa$ is the chirality parameter.

The focused beam is then represented as a superposition of plane waves corresponding to wavevectors ${\mathbf{k}}_{\sigma }(\theta ,\phi )$ with a fixed magnitude $k_{\sigma }=n_{\mathrm{m}}({\sigma })k_0$^39^:

14 $$\begin{aligned} {\mathbf {E}}_{\mathrm{in}}^{(\sigma )}({\mathbf {r}})= {} {E}_{0}\int _{0}^{2\pi }d\phi \int _{0}^{\theta _{0}}d{\theta }{{\rm sin}} \theta \, \sqrt{\cos \theta }\,e^{-\gamma ^2{{\rm sin}} ^2\theta } \, e^{i{\mathbf{k}}_{\sigma }(\theta ,\phi )\cdot ({\mathbf {r}}+{\mathbf {r}}_{p})}\,\hat{\varvec{\varepsilon }}_{\sigma }^{\prime }(\theta , \phi ). \end{aligned}$$

The polarization unit vector $\hat{\varvec{\varepsilon }}_{\sigma }^{\prime }(\theta , \phi ) =(\hat{\mathbf{x}}'+ i\,\sigma \, \hat{\mathbf{y }}')/\sqrt{2}$ is defined in terms of the Cartesian unit vectors obtained by rotation with Euler angles $(\phi ,\theta ,-\phi )$. The focal point is at position $-{\mathbf {r}}_{p}$, whereas the spherical particle center is at the origin^19^. After resolving for the Mie scattering by the particle and computing the resulting optical force, we displace the origin to the focal point, and then the particle position will be at ${\mathbf{r}}_p(\rho _p,\phi _p,z_p)$ which is finally written in terms of its cylindrical components. The angular semi-aperture $\theta _0$ is defined in terms of the objective NA as discussed in detail below, whereas $\gamma$ is the ratio of the objective focal length to the laser beam waist at the objective entrance port.

The incident focused beam (14) illuminates an achiral, non-magnetic spherical particle of refractive index $n_p=\sqrt{\varepsilon _p}$ and radius *a* which is embedded in the chiral medium. Mie scattering of a plane wave by a chiral spherical particle embedded in a chiral medium has been solved in Ref.^38^. We consider the particular case in which only the host medium is chiral. Our results differ from^63^ by some sign factors which we attribute to typos in that reference.

When considering the non-paraxial focused beam (14), we need to solve the Mie scattering for different propagation directions and then take the superposition of the corresponding scattered field components. Due to the spherical symmetry of the particle, it is straightforward to write the corresponding scattering field components with the help of finite rotations and Wigner rotation matrix elements $d_{m,m'}^{\ell }(\theta )$ in the angular momentum representation^64^.

The Debye potentials describing the total fields outside the spherical particle are written as partial-wave (multipole) sums over $\ell$ (for the total angular momentum $J^2$) and *m* (for the axial component $J_z$) of the form

$$\begin{aligned} \sum _{\ell ,m}\equiv \sum ^{\infty }_{\ell =1}\, \sum ^{\ell }_{m=-\ell }. \end{aligned}$$

Denoting the spherical coordinates of a spatial point ${\mathbf{r}}$ as $(r,\vartheta ,\varphi ),$ we find

15 $$\begin{aligned} \Pi ^{{\mathrm{E}}}(r,\vartheta ,\varphi )= {} i\sigma \frac{E_0}{k_\sigma } \sum _{\ell m} \gamma ^{(\sigma )}_{\ell ,m}\bigg [\, j_\ell (k_\sigma r ) + A_{\ell }\, h^{(1)}_\ell (k_\sigma r ) - \, B_{\ell } \, h^{(1)}_\ell (k_{-\sigma } r )\bigg ]Y_{\ell ,m}(\vartheta ,\varphi ) \end{aligned}$$

16 $$\begin{aligned} \Pi ^{{\mathrm{M}}}(r,\vartheta ,\varphi )= {} \sqrt{\frac{\varepsilon _m\varepsilon _0}{\mu _0}}\frac{E_0}{k_\sigma } \sum _{\ell m}\gamma ^{(\sigma )}_{\ell ,m} \bigg [\, j_\ell (k_\sigma r ) + A_{\ell }\, h^{(1)}_\ell (k_\sigma r ) +\, B_{\ell }\, h^{(1)}_\ell (k_{-\sigma } r )\bigg ]Y_{\ell ,m}(\vartheta ,\varphi ) \end{aligned}$$

Here, $j_{\ell }$ and $h^{(1)}_{\ell }$ denote the spherical Bessel and Hankel functions of the first kind, respectively, and $Y_{\ell ,m}$ are the spherical harmonics^65^. The multipole coefficients of the incident focused beam (14) are given by

17 $$\begin{aligned} \gamma ^{(\sigma )}_{\ell ,m}={} 2\pi \, i^{\ell -m+\sigma }\sqrt{\frac{4\pi (2{\ell }+1)}{{\ell }({\ell }+1)}}e^{-i(m-\sigma )\phi _p} \int _{0}^{\theta _{0}}d\theta {{\rm sin}} \theta \sqrt{\cos \theta }d^{\ell }_{m,\sigma }(\theta )J_{m-\sigma }(k_\sigma \rho _p{{\rm sin}} \theta _{}) e^{ik_\sigma z_p \cos \theta } \end{aligned}$$

where $J_{m}$ are the cylindrical Bessel functions of integer order *m*^65^. The scattering Mie coefficients $A_{\ell }\equiv \alpha _{\ell }/\Delta _{\ell }$ and $B_{\ell }\equiv \beta _{\ell }/\Delta _{\ell }$ represent the amplitudes for conserving and changing the photon helicity, respectively. They are given by

18 $$\begin{aligned} \alpha _{\ell }& = (N^{2}+1)\, \left[ \psi _\ell (x)\xi ^{\prime }_\ell ({\bar{x}})+ \xi _\ell ({\bar{x}}) \psi ^{\prime }_\ell (x) \right] \psi _\ell (y)\psi ^{\prime }_\ell (y) -2N\left[ \psi ^{\prime }_\ell (x) \xi ^{\prime }_\ell ({\bar{x}})\psi _\ell (y)^{2}+\psi _\ell (x) \xi _\ell ({\bar{x}})\psi ^{\prime }_\ell (y)^2 \right] \nonumber \\ \beta _{\ell }& = (N^{2}-1) \left[ \psi _\ell (x)\xi ^{\prime }_\ell (x) -\xi _\ell (x)\psi ^{\prime }_\ell (x)\right] \psi _\ell (y)\psi ^{\prime }_\ell (y) \nonumber \\ \Delta _{\ell }& = \left[ (N^{2}+1)\xi ^{\prime }_\ell (x)\psi _\ell (y) - 2N\xi ^{}_\ell (x)\psi ^{\prime }_\ell (y)\right] \xi _\ell ({\bar{x}})\psi ^{\prime }_\ell (y) +\left[ (N^{2}+1) \xi _\ell (x)\psi ^{\prime }_\ell (y) -2N \xi ^{\prime }_\ell (x) \psi _\ell (y)\right] \xi ^{\prime }_\ell ({\bar{x}}) \psi _\ell (y) \end{aligned}$$

here $N=n_p/n_{{\mathrm{m}}}(\sigma )$ is the relative refractive index of the particle with respect to the host medium and $\psi _\ell (x)=xj_\ell (x)$ and $\xi _\ell =xh^{(1)}_\ell (x)$ are the Riccati-Bessel functions^53^. The variables $x=n_{\mathrm{m}}(\sigma ) k_0 a$ and ${\bar{x}}=n_{{\mathrm{m}}}(-\sigma ) k_0 a$ are the size parameters in the host medium when taking the incident and the reversed helicities, respectively. Finally, $y=N\,x$ is the size parameter in the particle medium.

The coefficients $B_{\ell }$ appearing in Eqs. (15) and (16) represent amplitudes for helicity reversal $\sigma \rightarrow -\sigma$ upon Mie scattering. When the microsphere is aligned along the symmetry $z$-axis ($\rho _p=0$), the total optical angular momentum is conserved, and as a consequence the variation of SAM is entirely converted into optical orbital angular momentum^66^. Mie scattering is indeed a mechanism for spin-orbit interaction^67,68^. On the other hand, when $\rho _p>0,$ part of the SAM change might be transferred to the particle center-of-mass, thus contributing to the optical torque on the particle.

We now define the normalised force efficiency^17^

19 $$\begin{aligned} {\mathbf{Q}}= {\mathbf{F}}/(n_{{\mathrm{m}}}(\sigma ) P/c) \end{aligned}$$

where *P* is the laser power in the sample region and *c* is the speed of light in vacuum. When evaluating the flux of the stress tensor (13), we write the total electric field as

20 $$\begin{aligned} {\mathbf{E}}= {\mathbf{E}}^{(\sigma )}_{\mathrm{in}}+{\mathbf{E}}^{(\sigma )}_{{\mathrm{s}}}+{\mathbf{E}}^{(-\sigma )}_{{\mathrm{s}}} \end{aligned}$$

and likewise for the magnetic field $${\mathbf{H}}.$$$${\mathbf{E}}^{(\sigma )}_{{\mathrm{s}}}$$ ($${\mathbf{E}}^{(-\sigma )}_{{\mathrm{s}}}$$) represents the scattered field contribution with the same (opposite) helicity of the incident field. The three terms in (20) are ordered precisely as the three contributions in the r.-h.-s. of (15) and (16).

Among the several quadratic contributions obtained when replacing (20) into (13), cross terms involving opposite helicities do not contribute and only $$ {\mathbf{E}}^{(\sigma )}_{\mathrm{in}}\cdot {\mathbf{E}}^{(\sigma )}_{{\mathrm{s}}}{}^*,$$$${\mathbf{E}}^{(\sigma )}_{{\mathrm{s}}}\cdot {\mathbf{E}}^{(\sigma )}_{\mathrm{s}}{}^*$$ and $${\mathbf{E}}^{(-\sigma )}_{\mathrm{s}}\cdot {\mathbf{E}}^{(-\sigma )}_{{\mathrm{s}}}{}^*$$ survive when taking a Gaussian surface at infinity (and likewise for the terms quadratic in $${\mathbf{H}}$$ and the cross electric-magnetic terms). The first term yields the extinction contribution $${\mathbf{Q}}_{{\mathrm{e}}}$$ to the force efficiency, while the last two terms yield the scattering contribution $${\mathbf{Q}}_{{\mathrm{s}}}.$$$${\mathbf{Q}}_{{\mathrm{e}}}$$ represents the rate at which linear momentum is removed from the incident field, normalized as in (19). A fraction of this momentum is carried away by the scattered field at a normalized rate $$-{\mathbf{Q}}_{{\mathrm{s}}},$$ so that the total force efficiency is written as

21 $$\begin{aligned} {\mathbf{Q}}={\mathbf{Q}}_{{\mathrm{e}}}+{\mathbf{Q}}_{{\mathrm{s}}}. \end{aligned}$$

When deriving the multipole series for ${\mathbf{Q}}_{{\mathrm{e}}}$ and ${\mathbf{Q}}_{{\mathrm{s}}}$ from Eqs. (13) and (15)–(17), we introduce the effect of refraction at the planar interface between the glass coverslip and the chiral medium filling the sample, which is typical in oil-immersion objectives^69^. The refraction index mismatch between the two media is written in terms of the relative index $N_\sigma =n_{{\mathrm{m}}}(\sigma )/n_{{\mathrm{g}}},$ where $n_{{\mathrm{g}}}$ is the glass refractive index. The Fresnel amplitude for refraction is

$$\begin{aligned} T(\theta )= \frac{2\cos \theta }{\cos \theta +N_\sigma \cos \theta _{\mathrm{m}}} \end{aligned}$$

The wavevectors in glass have magnitude $k_{{\mathrm{g}}}=n_{{\mathrm{g}}}k_0$ and make an angle $\theta$ with respect to the *z*-axis, whereas the angle in the chiral medium is $\theta _{\mathrm{m}}=\arcsin ({{\rm sin}} \theta /N_\sigma ).$ The laser power *P* in the sample region is reduced on account of the interface, as well as from the finite aperture radius of the objective entrance port. The resulting filling fraction is given by

$$\begin{aligned} F_{\sigma } = 16\gamma ^2\int _0^{s_0} ds\,s\,e^{(-2\gamma ^2s^2)}\,\frac{\sqrt{(1-s^2)(N_{\sigma }^2-s^2)}}{\left( \sqrt{1-s^2}+\sqrt{N_{\sigma }^2-s^2}\right) ^2}, \end{aligned}$$

with $$s_0=\min \{N_{\sigma },\text{ NA }/n_{{\mathrm{g}}}\}$$.

More importantly, we add to the ideal aplanatic model (14) the spherical aberration phase^70^

22 $$\begin{aligned} \Phi _{{\mathrm{sa}}}(\theta )=k_{{\mathrm{g}}}\left( -L/N_{\sigma }\cos \theta +N_{\sigma }L\cos \theta _{{\mathrm{m}}}\right) , \end{aligned}$$

also introduced by refraction at the glass-sample interface. Spherical aberration is typically detrimental on the effects discussed in this paper as it degrades the focal region. Thus, our realistic description of oil-immersion objectives, which are usually employed in optical tweezers setups, prevents us from overestimating the optical torque discussed in the following. The phase $\Phi _{\mathrm{sa}}(\theta )$ is proportional to the distance *L* between the glass slide and paraxial focal plane. Instead of assuming a given value for *L*,  which is unknown in real experiments, we simulate the experimental procedure for controlling the amount of spherical aberration (see Methods). The relevant length scale is then the distance *d* by which the objective is displaced, starting from the configuration with the trapped microsphere just touching the coverslip.

The axial components of ${\mathbf{Q}}_{{\mathrm{s}}}$ and ${\mathbf{Q}}_{{\mathrm{e}}}$ are then written as

23 $$\begin{aligned} Q_{s z}= {} -\frac{16\gamma ^2}{F_{\sigma }N_{\sigma }}{\mathrm{Re}}\Biggl \{\sum _{\ell m}\frac{\sqrt{\ell (\ell +2)(\ell +m+1)(\ell -m+1)}}{\ell +1} \biggl [\biggl (1+\frac{\sigma \,\kappa }{n_{{\mathrm{m}}}}\biggr )A_{\ell }A_{\ell +1}^{*}+\biggl (1-\frac{\sigma \,\kappa }{n_{{\mathrm{m}}}}\biggr )B_{\ell }B_{\ell +1}^{*}\biggr ] \nonumber \\&\times G^{(\sigma )}_{\ell ,m}G^{(\sigma )*}_{\ell +1,m} +\frac{1}{2} \frac{(2\ell +1)}{\ell (\ell +1)}\,m\,\sigma \, \biggl [\biggl (1-\frac{\sigma \,\kappa }{n_{{\mathrm{m}}}}\biggr )\vert A_{\ell }\vert ^2-\biggl (1+\frac{\sigma \,\kappa }{n_{\mathrm{m}}}\biggr )\vert B_{\ell }\vert ^2\biggr ]\vert G^{(\sigma )}_{\ell ,m}\vert ^2\Biggr \}, \end{aligned}$$

24 $$\begin{aligned} Q_{ez}= {} \frac{8\gamma ^2}{F_{\sigma }N_{\sigma }}{\mathrm{Re}}\sum _{\ell m}(2\ell +1) \biggl (1-\sigma \frac{(i-1)}{2}\frac{\kappa }{n_{\mathrm{m}}}\biggr )\,A_{\ell }\,G^{(\sigma )}_{\ell ,m}\,G'^{(\sigma )*}_{\ell ,m}, \end{aligned}$$

Like the axial components, the radial and azimuthal cylindrical components of ${\mathbf{Q}}_{{\mathrm{s}}}$ and ${\mathbf{Q}}_{{\mathrm{e}}}$ do not depend on the particle angular position $\phi _p$ by symmetry (see Methods). The dependence on the cylindrical coordinates $\rho _p$ and $z_p$ of the particle position is contained in the multipole coefficients

25 $$\begin{aligned} G_{\ell m}^{(\sigma )}(\rho _p,z_p)=\int _{0}^{\theta _0}d\theta {{\rm sin}} \theta \sqrt{\cos \theta }\,T(\theta )\, e^{-\gamma ^2{{\rm sin}} ^2\theta } d_{m,\sigma }^{\ell }(\theta _{{\mathrm{m}}})\, J_{m-\sigma }\left( k_{{\mathrm{g}}} \rho _p{{\rm sin}} \theta \right) e^{i[\Phi _{{\mathrm{sa}}}(\theta )+k_{\sigma }\cos \theta _{{\mathrm{m}}} z_p ]} \end{aligned}$$

26 $$\begin{aligned} G'^{(\sigma )}_{\ell ,m}(\rho _p,z_p)=\int _{0}^{\theta _0}d\theta {{\rm sin}} \theta \cos \theta _{{\mathrm{m}}} \sqrt{\cos \theta }\,T(\theta )\,e^{-\gamma ^2{{\rm sin}} ^2\theta }d_{m,\sigma }^{\ell }(\theta _{\mathrm{m}})\, J_{m-\sigma }\left( k_{{\mathrm{g}}}\rho _p {{\rm sin}} \theta \right) e^{i[\Phi _{{\mathrm{sa}}}(\theta )+k_{\sigma }\cos \theta _{{\mathrm{m}}} z_p ]} \end{aligned}$$

The upper bound for the integration in (25) and (26) represents the angular semi-aperture in the glass medium: $\theta _{0}={{\rm sin}} ^{-1}\{{\mathrm{min}}[({\mathrm{NA}}/n_{\mathrm{g}}),N_\sigma ]\}.$ We do not take evanescent waves (which appear when ${\mathrm{NA}}> n_{{\mathrm{m}}}(\sigma )$) into account^71^ as the microsphere is trapped near the focal plane and thus far from the coverslip for the typical numerical examples discussed below.

### Numerical examples

In all numerical examples discussed in this paper, we take typical experimental values for a standard optical tweezers setup^20^. We consider a right-handed CP Gaussian beam ($\sigma =-1$) with vacuum wavelength $\lambda _0=1064\,{\mathrm{nm}}$ at the objective entrance port, of numerical aperture $\hbox {NA}=1.3.$ Results for left-handed CP ($\sigma =1$) may be considered from those shown here by replacing $\kappa \rightarrow -\kappa$ and changing the sign of the azimuthal force component (and its derivative). The ratio between the objective focal length and the beam waist at the entrance port is $\gamma =1.226.$ The objective axial displacement, which controls the amount of spherical aberration when employing oil-immersion objectives, is $d=5$ μm. In addition, we take $n_{{\mathrm{g}}}=1.5$ and $\varepsilon _{{\mathrm{m}}}= 1.85$ for the refractive index of the glass coverslip and the permittivity of the chiral solution, respectively.

#### Optical force

**Figure 1:**
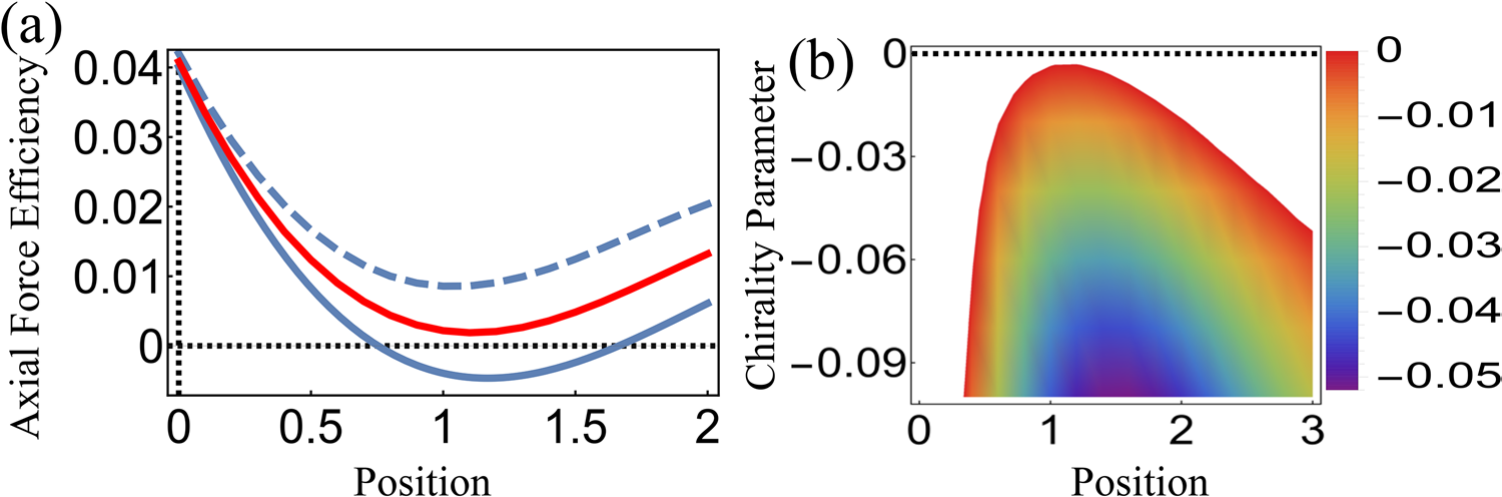
(**a**) Normalized axial force $Q_z$ acting on a $\hbox {BrO}_2$ microsphere of radius $500\,{\mathrm{nm}}$ embedded in a chiral medium as a function of axial position (in units of the sphere radius) for different chirality parameters: $\kappa = -0.01$ (solid), $\kappa$ = 0 (red) and $\kappa = 0.01$ (dashed). The incident beam is right-handed circularly polarized (helicity $\sigma =-1$). (**b**) Density plot of $Q_z$ versus axial position and chirality parameter. Only negative values are shown.

As a first example, we consider a $\hbox {BrO}_2$ microsphere with refractive index $n_p=1.7$ and radius $a = 500\, {\mathrm{nm}}$ immersed in a chiral solution. In Fig. 1a, we plot the axial force efficiency $Q_z$ as a function of normalized microsphere position $z_p/a$ along the $z$-axis ($\rho _p=0$) for different values of chirality parameter $\kappa$. When the host medium is achiral ($\kappa =0$), its refractive index $n_{{\mathrm{m}}}=\sqrt{\varepsilon _{{\mathrm{m}}}}=1.36$ is too small compared to the $\hbox {BrO}_2$ particle’s high refractive index. As a consequence, radiation pressure dominates, leading to a positive (i.e. along the propagation direction) force for all values of $z_P/a$ (red line).

In contrast, trapping can be achieved in a chiral host media with $\kappa =-0.01$ under otherwise the same conditions, as indicated by the solid blue line in Fig. 1a. Thus, a chiral medium with the same handedness of the CP trapping laser beam allows for trapping of high-index particles by diminishing the radiation pressure effect and leading to negative optical forces. On the other hand, in the case of a left-handed chiral medium $\kappa _m=0.01$ (dashed line), radiation pressure is enhanced and again no trapping is possible.

Figure 1b shows the density plot of the axial force efficiency $Q_z$ as a function of the chirality parameter $\kappa$ and the axial position $z_p/a.$ The colored area corresponds to the regions in the parameter space for which the optical force is negative ($Q_z<0$), thus allowing for stable trapping. The edge of this area provides the positions of equilibria along the *z*-axis as function of $\kappa ,$ with the left-hand side corresponding to stable equilibria. It is worthwhile to mention that chiral media not only optimize trapping stability but also facilitate optical tweezing of large refractive-index particles, as in the example considered here. In short, trapping in a chiral host media facilitates optical manipulation and tweezing of high-index particles provided that the chiral material has the same handedness of the incident CP light.

#### Optical torque

Spin angular momentum (SAM) of CP light can be transferred to trapped particles and make them spin around the beam axis when they are absorptive, anisotropic^22^ or non-spherical^23^. Although the optical torque (OT) on a transparent isotropic microsphere centered along the beam symmetry axis vanishes, transfer of SAM to the center of mass can still be observed in this case from the analysis of Brownian fluctuations^24^ or by driving the sample laterally so as to displace the equilibrium position from the beam axis^26^. OT is predicted to be significantly enhanced in the case of chiral particles, opening the way for enantioselective manipulation and characterization of the chiral response of individual nanoparticles with optical tweezers^36^.

**Figure 2:**
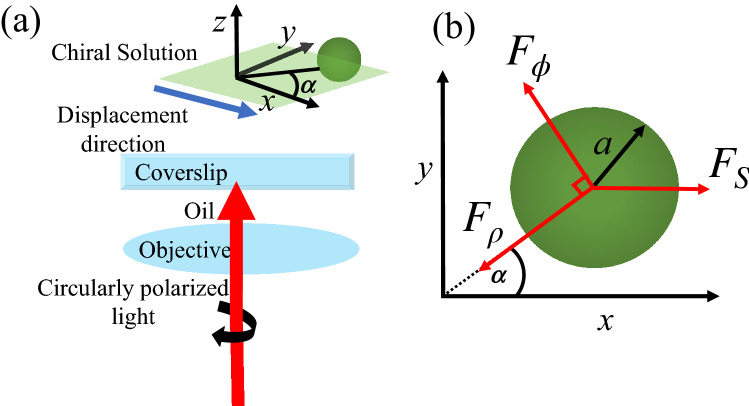
(**a**) Schematic representation of the optical torque on a particle trapped in a chiral medium. A right-handed ($\sigma =-1$) circularly-polarized Gaussian laser beam is focused by an oil-immersion high-NA objective into a sample filled with a chiral solution. The sample is driven laterally so as to displace the particle equilibrium position from the beam symmetry axis. (**b**) At equilibrium, the resulting Stokes drag force $F_S$ balances the optical force, which contains radial $F_{\rho }$ and azimuthal $F_{\phi }$ components, the latter being responsible for the optical torque. The equilibrium position is then rotated around the beam axis by an angle $\alpha$ with respect to the direction of the Stokes force.

Here we show that a much stronger enhancement of the OT is found when taking a chiral host medium instead of a chiral particle. We follow the scheme of Ref.^26^ and calculate the rotation of the equilibrium position when a Stokes drag force is applied by driving the sample along the $$x$$-direction, as illustrated by Fig. 2a. As the particle is displaced off-axis by the Stokes force $$F_S$$, an optical azimuthal component $$F_{\phi }$$ builds up, in addition to the restoring radial component $$F_{\rho }<0.$$$$F_\phi $$ results from the SAM of the trapping beam and its sign is controlled by the helicity $$\sigma $$ of the CP. As shown in Fig. 2b, the resulting equilibrium position is then rotated by an angle $$\alpha $$ around the $$z$$-axis with respect to the $$x$$-axis, with $$\tan \alpha = F_\phi /|F_\rho |.$$ When the off-axis displacement is $$\ll a,$$ we can write the rotation angle in terms of the transverse optical stiffness $$k_{\rho }\equiv -\partial _\rho F_\rho |_{\rho =0}$$ and the torsion constant $$k_{\phi }\equiv \partial _\rho F_\phi |_{\rho =0}$$ as $$\tan \alpha \approx k_{\phi }/k_{\rho }.$$ We obtain exact values for $$k_\phi $$ and $$k_\rho $$ from the Mie-Debye theory for chiral host media developed above. We first derive partial-wave series for $$k_\phi $$ and $$k_\rho $$ by taking the analytical derivatives of the series for $$Q_\phi $$ and $$Q_\rho .$$ The series for $$k_\phi $$ and $$k_\rho $$ are then computed numerically. By rotational symmetry, they are independent of the angular position $$\alpha .$$

**Figure 3:**
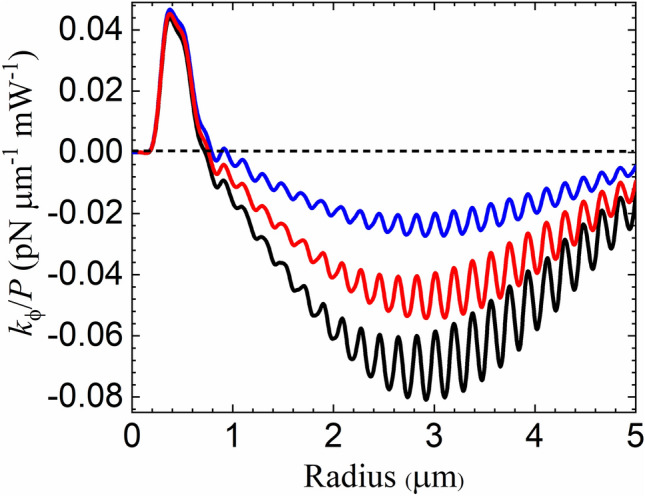
Torsion constant per unit power $k_{\phi }/P$ as a function of the radius of a silica microsphere. The particle is embedded in a chiral solution with $\kappa =-0.001$ (blue), $-0.002$ (red) and $-0.003$ (black). The incident beam is right-handed circularly-polarized (helicity $\sigma =-1$).

In all numerical examples for the optical torque, we consider a silica microsphere with refractive index $n_p = 1.46$ embedded in a chiral medium. In Fig. 3, we plot $k_{\phi }/P$ as a function of the sphere radius *a* for chirality parameters $\kappa =-0.001$ (blue), $-0.002$ (red) and $-0.003$ (black). For radii $a{\mathop {\scriptscriptstyle \sim }\limits ^{<}} \lambda _0/n_m(\sigma ),$ the torsion constant is approximately independent of $\kappa$ and develops a peak corresponding to a negative torque^72^, i.e, opposite to the SAM of the trapping beam, at $a\approx 0.4$ μm. As the radius increases, the OT goes positive and $k_\phi$ develops a second (negative) peak at $a\approx 2.9$ μm, whose amplitude is strongly chirality-dependent. For even larger radii (not shown), $k_\phi$ oscillates around zero as expected in the geometrical optics regime^19^. The oscillations result from interference between direct reflection and reflection after a round-trip propagation across the microsphere diameter^18^. Such interference oscillations, of period $\Delta a= \lambda _0/(4n_p)\approx 0.18$ μm, are clearly visible in the negative peak shown in Fig. 3.

The spin-orbit contribution to $k_\phi$ can be traced by collecting the spin-reversal terms involving the coefficients $B_{\ell }$ as discussed in connection with Eqs. (15) and (16). In the peak around $a\approx 0.4$ μm shown in Fig. 3, the spin-orbit contribution is negative and its magnitude varies in the range between 15 and 20% of the total result. It becomes more dominant for smaller particles, closer to the Rayleigh scattering regime, for which $k_\phi$ becomes negligibly small and $\kappa -$independent. On the other hand, the spin-orbit effect accounts for a small fraction of the torsion constant $k_\phi ,$ typically at the percent level, near the chirality-dependent peak around $a\approx 2.9$ μm. Overall, the relative contribution of the spin-orbit term tends to decrease with the chirality parameter $\kappa .$

**Figure 4:**
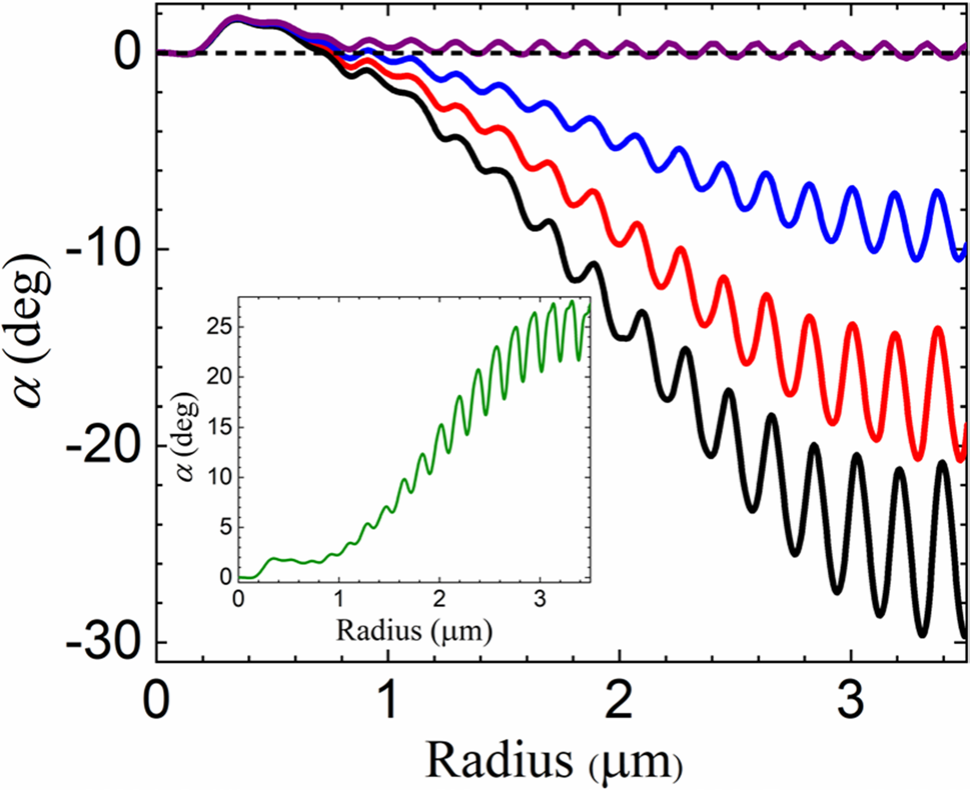
Microsphere rotation angle $\alpha$ in degrees resulting from the optical torque on a silica microsphere (see Fig. 2) as a function of radius. The chirality parameter of the host medium is $\kappa =0$ (purple), $-0.001$ (blue), $-0.002$ (red) and $-0.003$ (black). The inset shows the case of a left-handed host medium with $\kappa =0.003$ (green) for comparison. The incident beam is right-handed circularly-polarized (helicity $\sigma =-1$). The incident beam is right-handed circularly-polarized (helicity $\sigma =-1$).

Such chirality-dependent enhancement of the OT illustrated by Fig. 3 leads to a significant increase of the rotation angle $\alpha .$ In Fig. 4, we plot $\alpha$ as a function of sphere radius, again for different values of the chirality parameter (same conventions as in Fig. 3). We also show the case of an achiral medium ($\kappa =0$, purple), for which the rotation is significant only for radii near $a\sim 0.4$ μm, resulting from a negative OT recently measured for polystyrene microspheres^26^. For chiral media, the magnitude of the rotation angle is maximum at $a\approx 3.4$ μm, which is slightly shifted with respect to the peak position of $k_{\phi }$ because the transverse stiffness decays as $k_\rho \sim 1/a$ in this size range^19^. The behavior of $k_\rho$ also explains why the ratio between the amplitudes of the two peaks for the angle of rotation is much bigger than the corresponding ratio for the torsion constant $k_\phi$ shown in Fig. 3.

The interference oscillations discussed in connection with Fig. 3 are also clearly visible in the plot of the rotation angle shown in Fig. 4. The fast, large-amplitude oscillations near the peak region open the way for measurements of the microsphere radius. For $\kappa = -0.003,$ the oscillations correspond to a maximum slope $\Delta \alpha /\Delta a \sim 130^{\mathrm{o}}\mu{\mathrm{m}}^{-1}$ near the peak region, allowing for a sensitivity $\delta a\sim 1.5\,{\mathrm{nm}}$ given a typical conservative estimate $\delta \alpha \sim 0.2^{{\mathrm{o}}}$ for the precision in the measurement of the rotation angle^26^.

The inset of Fig. 4 shows the case of a left-handed chiral host medium with $\kappa =0.003.$ As the handedness of the medium is opposite to the handedness of the incident trapping beam, the OT is always negative. Although the peak value is slightly smaller than the magnitude of the peak for $\kappa =-0.003,$ it still corresponds to a remarkable enhancement of the negative OT effect when compared with the experiment reported in^26^.

**Figure 5:**
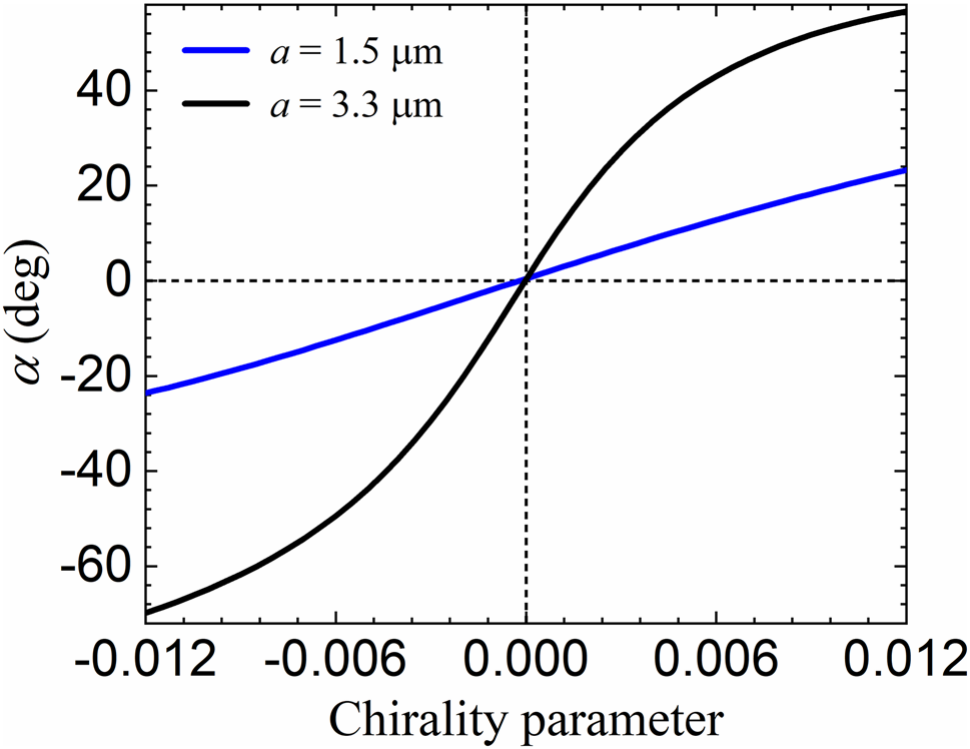
Microsphere rotation angle $$\alpha $$ in degrees versus chirality parameter $$\kappa $$ of the host medium for a silica microsphere of radius $$a=1.5 $$ μm (blue) and $$3.3$$ μm (black). The incident beam is right-handed circularly-polarized (helicity $$\sigma =-1$$).

The comparison between the results for opposite signs of $$\kappa $$ shown in Fig. 4 shows that the sense of rotation can be employed as a direct probe of the handedness of the medium when using microspheres of radii $$a>1$$ μm. The angle $$\alpha $$ indeed changes sign as $$\kappa $$ changes from negative to positive values as illustrated by Fig. 5, where we plot the rotation angle $$\alpha $$ as a function of the chirality parameter $$\kappa $$ for $$a=1.5 $$ μm (blue) and $$3.3$$ μm (black).

The strong dependence of the rotation angle on the chirality parameter illustrated by Fig. 5 paves the way for an all-optical, local characterization of the host medium chiral response at the nanoscale with the help of optical tweezers. For the radius $a=3.3$ μm, the slope of the function $\alpha (\kappa )$ in the neighborhood of $\kappa =0$ is $\Delta \alpha /\Delta \kappa \approx 9.9\times 10^3\,{\mathrm{deg}},$ thus allowing for a chirality resolution $\delta \kappa \sim 2\times 10^{-5}$ given a typical experimental precision $\delta \alpha \sim 0.2^{{\mathrm{o}}}.$ Such figures bring naturally occurring chiral solutions within reach of our proposal for characterization of chirality, which seems to be ideally suited for the small-volume microfluidic chambers often employed as samples in optical tweezers setups^8^.

## Discussion

We have shown that the optical force acting on a dielectric trapped microsphere embedded in a chiral medium strongly depends on the chirality parameter $\kappa$ and on the handedness of the CP trapping beam. The trap axial stability is greatly enhanced by choosing a CP beam with the same handedness of the host medium. Such arrangement allows for optical tweezing of high-refractive index particles that cannot be trapped otherwise, thus enlarging the scope of single-beam optical traps.

We have also considered the optical torque on the trapped particle’s center of mass, which is characterized by the torsion constant $k_\phi ,$ in order to unveil the remarkable interplay between chirality and the transfer of optical spin angular momentum.

Our approach allows for a clear identification of the spin-orbit contribution to the optical force and torque. Mie scattering of a CP incident field gives rise to a field component with the reserved SAM^66,67^. Such spin-to-orbit conversion becomes more transparent in the formalism developed in this paper, as field components with opposite helicities propagate with different phase velocities in a chiral host medium. The spin-orbit effect provides a sizable contribution for radii of the order of the wavelength and increases as the radius is decreased into the Rayleigh scattering regime.

The optical torque leads to a rotation of the equilibrium position when a lateral external Stokes force is applied. We have found a sizeable, detectable enhancement of the rotation angle for radii $a\sim 3$ μm for media with chiral indices compatible with those of naturally occurring materials. Since the angle depends strongly on the chirality parameter in this range of radii, one might characterize the local chiral response of small-volume samples typically employed in optical tweezers from measurements of the equilibrium position similar to those reported in Ref.^26^. In addition, the sense of rotation provides a direct indication of the handedness of the solution. Altogether our findings show that the torque in optical tweezers could be exploited as a novel all-optical method to locally probe the chiral response at the nanoscale. It is important to distinguish this method from traditional optical methods of enantioselection of chiral solutions, such as the rotatory power, which only apply for macroscopically large systems and can only provide an average chiral response.

When considering the torsion constant in a chiral host medium, the geometrical optics result is obtained only for radii much larger than usually required. We have obtained interference oscillations which are typical for radii larger than the wavelength^19^. They arise from an unusual type of semiclassical Mie scattering near the focal region, with the leading contribution coming from small angular momenta (small multipole orders)^18^. We have found oscillation amplitudes much larger than the typical values for achiral materials^69^ when considering radii close to $a\sim 3$ μm. Such oscillations open the way for the characterization of the microsphere diameter with nanometric precision. On the other hand, from a more fundamental perspective, it brings into light an unexpected feature of semiclassical Mie scattering^73^ that requires further investigation.

## Methods

### Numerical simulation of the spherical aberration introduced by the glass-sample interface

**Figure 6:**
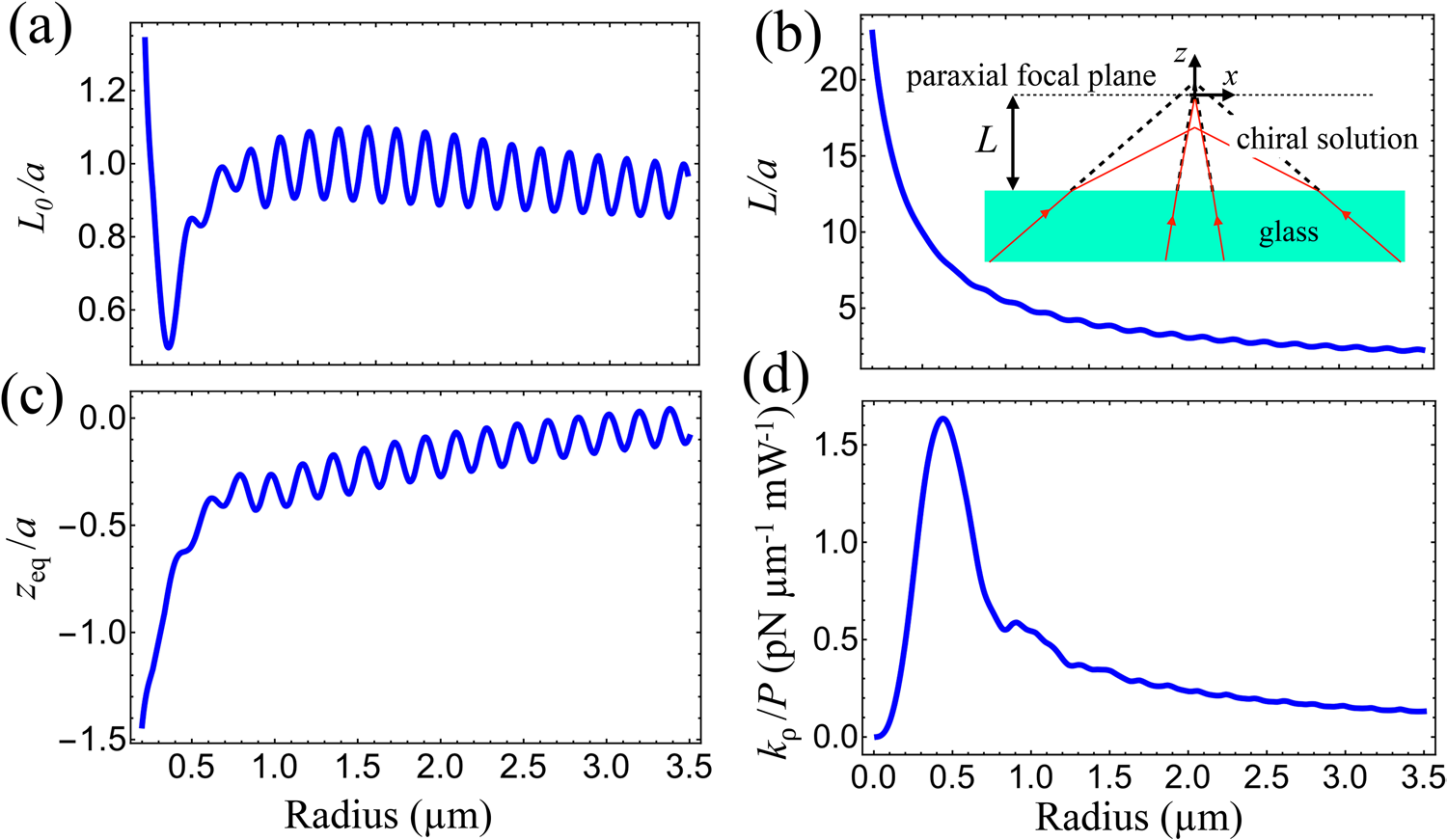
Numerical simulation of the effect of refraction at the planar interface between the glass coverslip and the interior of the sample chamber. As an example, we take $$\kappa =-0.003.$$ (**a**) Initial reference position of the focal plane with respect to the glass slide versus radius. The reference configuration is defined by the condition that the equilibrium position is such that the microsphere is just touching the glass slide. (**b**) Final focal plane position (in units of sphere radius) after displacing the objective by $$d= 5 $$ μm. (**c**) Equilibrium position of the microsphere (in units of sphere radius) and (**d**) transverse trap stiffness $$k_{\rho }$$ (in units of power).

In real experiments, the distance *L* between the paraxial focal plane and the planar interface between the glass coverslip and the sample chamber (see inset of Fig. 6b) is not known beforehand. In order to control this parameter, which defines the amount of spherical aberration according to Eq. (22), one can start from a reference configuration with the trapped microsphere just touching the coverslip, which is easy to identify experimentally^69^. Then, the objective is displaced away from the coverslip so as to trap the particle at a comfortable distance from the boundary of the chamber.

Here, we describe how we simulate such experimental procedure numerically. To illustrate the method, we show explicit intermediate results for silica microspheres embedded in a chiral solution with $\kappa = -0.003.$ All other examples discussed in the paper are obtained along the same lines.

We first calculate the interface-focal plane distance $L_0$ in the initial reference configuration, using the condition $z_{\mathrm{eq}}^{(0)}+L_0=a,$ where $z_{{\mathrm{eq}}}^{(0)}$ is the particle equilibrium position (measured with respect to the focal plane) in the reference configuration. Thus, we solve $Q_z(z_p=a-L_0)=0$ for $L_0$ as a function of radius. The results are shown in Fig. 6a.

The second step is to increase *L* by a controlled amount: $L=L_0+N_\sigma d,$ where *d* is the objective displacement. In Fig. 6b we plot *L*/*a* versus radius for $d=5$ μm. Once the coverslip-focal plane distance is known, one can either directly compute the axial force as a function of the axial position $z_p$ (see Fig. 1), or continue the procedure by solving $Q_z(z_p=z_{{\mathrm{eq}}})=0$ for the stable equilibrium position $z_{\mathrm{eq}}.$ The results for $z_{{\mathrm{eq}}}$ as a function of radius, shown in Fig. 6c, display interference oscillations with a characteristic period $\Delta a = \lambda _0/(4n_{{\mathrm{s}}})$ already discussed in connection with Fig. 3.

Finally, the last step consists in computing the partial-wave (multipole) series for $k_\phi$ and $k_\rho$ taking $\rho _p=0$ and $z_p=z_{{\mathrm{eq}}}.$ The results for $k_\phi /P$ and $k_\rho /P$ are shown in Fig. 3 (black line) and Fig.  6d, respectively. Note that $k_\rho >0$ for all radii as required for trap stability on the *xy* plane.

### Multipole series for the radial and azimuthal force components

The axial components of the scattering and extinction optical force components, normalized by Eq. (19), are given by (23) and (24), respectively. Here, we provide the remaining cylindrical components.

- Scattering radial component
  27 $$\begin{aligned}Q_{s\rho }& =-\frac{8\gamma ^2}{F_{\sigma }N_{\sigma }} {\mathrm{Im}}\left\{\sum _{\ell m} \frac{(2\ell +1)}{\ell (\ell +1)}\sqrt{(\ell -m)(\ell +m+1)}\,\sigma \, \left[\left(1-\frac{\sigma \, \kappa }{n_{{\mathrm{m}}}}\right)\vert A_{\ell }\vert ^2-\left(1+\frac{\sigma \, \kappa }{n_{\mathrm{m}}}\right)\vert B_{\ell }\vert ^2\right] \nonumber \right.\\&\left.\quad \times G^{(\sigma )}_{\ell ,m}G^{(\sigma )*}_{\ell ,m+1} - \frac{\sqrt{\ell (\ell +2)(\ell +m+1)(\ell +m+2)}}{\ell +1} \left[\left(1+\frac{\sigma \, \kappa }{n_{\mathrm{m}}} \right)A_{\ell }A_{\ell +1}^{*}+\left(1-\frac{\sigma \, \kappa}{n_{{\mathrm{m}}}}\right)B_{\ell }B_{\ell +1}^{*}\right]\right.\nonumber \\&\left.\quad \times \left( G^{(\sigma )}_{\ell ,m}G^{(\sigma )*}_{\ell +1,m+1} +G^{(\sigma )}_{\ell ,-m}G^{(\sigma )*}_{\ell +1,-(m+1)}\right) \right\} \end{aligned}$$
- Scattering azimuthal component
  28 $$\begin{aligned}Q_{s\phi }& = \frac{8\gamma ^2}{F_{\sigma }N_{\sigma }}{\mathrm{Re}}\left \{\sum _{\ell m } \frac{(2\ell +1)}{\ell (\ell +1)}\sqrt{(\ell -m)(\ell +m+1)}\,\sigma \, \left [\left (1-\frac{\sigma \, \kappa }{n_{{\mathrm{m}}}}\right )\vert A_{\ell }\vert ^2-\left (1+\frac{\sigma \, \kappa }{n_{\mathrm{m}}}\right )\vert B_{\ell }\vert ^2\right ] \nonumber \right.\\&\left.\quad \times G^{(\sigma )}_{\ell ,m}G^{(\sigma )*}_{\ell ,m+1} - \frac{\sqrt{\ell (\ell +2)(\ell +m+1)(\ell +m+2)}}{\ell +1} \left [\left (1+\frac{\sigma \, \kappa }{n_{\mathrm{m}}}\right )A_{\ell }A_{\ell +1}^{*}+\left (1-\frac{\sigma \,\kappa }{n_{\mathrm{m}}}\right )B_{\ell }B_{\ell +1}^{*}\right ]\nonumber \right.\\&\left.\quad \times \left( G^{(\sigma )}_{\ell ,m}G^{(\sigma )*}_{\ell +1,m+1} -G^{(\sigma )}_{\ell ,-m}G^{(\sigma )*}_{\ell +1,-(m+1)}\right) \right\} \end{aligned}$$
- Extinction radial component
  29 $$\begin{aligned} Q_{e\rho }(\rho ,\phi ,z)=\frac{4\gamma ^2}{F_{\sigma }N_{\sigma }}{\mathrm{Im}}\sum _{\ell m}(2\ell +1) \biggl (1-\sigma \frac{(i-1)}{2}\frac{\kappa }{n_{{\mathrm{m}}}}\biggr ) A_{\ell }\, G^{(\sigma )}_{\ell ,m} \, \left( G^{(\sigma )-}_{\ell ,m+1} - G^{(\sigma )+}_{\ell ,m-1}\right) ^* \end{aligned}$$
- Extinction azimuthal component
  30 $$\begin{aligned} Q_{e\phi }(\rho ,\phi ,z)=-\frac{4\gamma ^2}{F_{\sigma }N_{\sigma }}{\mathrm{Re}}\sum _{\ell m}(2\ell +1) \biggl (1-\sigma \frac{(i-1)}{2}\frac{\kappa }{n_{{\mathrm{m}}}}\biggr ) \,A_{\ell }\,G^{(\sigma )}_{\ell ,m}\, \left( G^{(\sigma )+}_{\ell ,m-1}+G^{(\sigma )-}_{\ell ,m+1}\right) ^* \end{aligned}$$

The multipole coefficients $G_{\ell m}^{(\sigma )}$ are given by Eq. (25). The extinction radial and azimuthal components also require the coefficients

31 $$\begin{aligned} G_{\ell ,m}^{(\sigma )\pm }(\rho _p,z_p)=\int _{0}^{\theta _0}d\theta {{\rm sin}} \theta {{\rm sin}} \theta _{{\mathrm{m}}} \sqrt{\cos \theta }\,T(\theta )\, e^{-\gamma ^2{{\rm sin}} ^2\theta }d_{m\pm 1,\sigma }^{\ell }(\theta _{{\mathrm{m}}})\, J_{m-\sigma }\left( k_{{\mathrm{g}}} \rho _p{{\rm sin}} \theta \right) e^{i[\Phi _{\mathrm{sa}}(\theta )+k_{\sigma }\cos \theta _{{\mathrm{m}}} z_p ]}, \end{aligned}$$

One can show that the above results, alongside Eqs. (23) and (24) for the axial components, are such that

$$\begin{aligned} (Q_z,Q_\rho ,Q_\phi )\rightarrow (Q_z,Q_\rho ,-Q_\phi ) \end{aligned}$$

when taking $(\sigma \rightarrow -\sigma ,\, \kappa \rightarrow -\kappa )$ as expected for the cylindrical components of a polar vector.
